# Dutch normative data of the sexual distress scale and the body image scale

**DOI:** 10.1007/s11136-023-03434-w

**Published:** 2023-05-16

**Authors:** Anouk S. Huberts, Noëlle J. M. C. Vrancken Peeters, Z. L. Rana Kaplan, Reinier C. A. van Linschoten, H. Pastoor, C. Janneke van der Woude, Linetta B. Koppert

**Affiliations:** 1 Department of Quality and Patientcare, Erasmus University Medical Center, 3015 GD Rotterdam, The Netherlands; 2 Academic Breast Cancer Center, Department of Surgical Oncology, Erasmus MC Cancer Institute, Erasmus University Medical Center, Rotterdam, The Netherlands; 3 Department of Public Health, Erasmus University Medical Center, Rotterdam, The Netherlands; 4 Department of Gastroenterology & Hepatology, Franciscus Gasthuis & Vlietland, Rotterdam, The Netherlands; 5 Department of Gastroenterology & Hepatology, Erasmus Medical Center, Rotterdam, The Netherlands; 6 Division of Reproductive Medicine, Department of Obstetrics and Gynecology, Erasmus University Medical Center, Rotterdam, The Netherlands

**Keywords:** Sexual wellbeing, Sexual distress, Body image, Patient reported outcome measure, Value based healthcare

## Abstract

**Purpose:**

Sexual health is an important contributing factor for health-related quality of life, but research in this domain is scarce. Moreover, normative data are needed to interpret patient-reported outcome measures on sexual health. The aim of this study was to collect and describe normative scores of the Female Sexual Distress Scale (FSDS) and the Body Image Scale (BIS) from the Dutch population and assess the effect of important demographic and clinical variables on the outcome. As the FSDS is also validated in men, we refer to it as SDS.

**Method:**

Dutch respondents completed the SDS and BIS between May and August 2022. Sexual distress was defined as a SDS score > 15. Descriptive statistics were calculated to present normative data per age group per gender after post-stratification weighting was applied. Multiple logistic and linear regression analyses were conducted to assess the effect of age, gender, education, relationship status, history of cancer and (psychological) comorbidities on SDS and BIS.

**Results:**

For the SDS 768 respondents were included with a weighted mean score of 14.41 (SD 10.98). Being female (OR 1.77, 95% CI [1.32; 2.39]), having a low educational level (OR 2.02, CI [1.37; 2.39]) and psychological comorbidities (OR: 4.86, 95% CI [2.17; 10.88]) were associated with sexual distress. For the BIS, 696 respondents were included. Female gender (β: 2.63, 95% CI [2.13; 3.13]), psychological comorbidities (β: 2.45, 95% CI [1.43; 3.47]), higher age (β: −0.07, 95% CI [−0.09; −0.05]), and a high educational level (β:−1.21, CI: −1.79 to −0.64) were associated with the non-disease related questions of the Body Image Scale.

**Conclusion:**

This study provides age- and gender-dependent normative values for the SDS and the non-disease related questions of the BIS. Sexual distress and body image are influenced by gender, education level, relationship status and psychological comorbidities. Moreover, age is positively associated with Body Image.

**Supplementary Information:**

The online version contains supplementary material available at 10.1007/s11136-023-03434-w.

## Introduction

Survival rate and life expectancy have improved in numerous diseases. This leads to an increasing number of patients living with long-term consequences of diseases which affect different domains of their health-related quality of life (HRQoL) [1, 2]. Sexual health is an important contributing factor for HRQoL [3, 4], but research in this domain is scarce and mostly focuses on sexual functioning rather than sexual well-being [4–9]. After all, sexual health is not merely the absence of disease, dysfunction or infirmity, but is defined as a state of physical, emotional, mental and social wellbeing in relation to sexuality [10]. Several studies showed that the majority of people who reported difficulties in sexual function report no or minimal sexual distress [11–13]. Additionally, these studies reported that sexual functioning and sexual distress are related to different risk factors. These finding address the importance of investigating both sexual domains [13–15].

The Female Sexual Distress Scale (FSDS) and Body Image Scale (BIS) are two patient-reported outcome measures which address different aspects of sexual health and could be supplementary to questionnaires about sexual function [16–19].

However, normative scores are needed to interpret these patient-reported outcome measures, to provide context of individual scores in the consultation room, and enable future comparison between people with a specific disease and the general Dutch population. Normative data describes outcomes of a defined population without the specific condition of interest [20]. In this study we aim to collect and describe normative scores of the Female Sexual Distress Scale and the Body Image Scale from the Dutch population and assess the effect of clinical important variables on the outcome. As the FSDS is also validated in men, we refer to it as SDS and will assess the sexual distress in both men and woman.

## Methods

### Patient-reported outcome measures (PROM)

The SDS is a 12-item scale; each question is scored on a 4-point Likert scale. For analyzing all the items must be added, with the maximum score of 48. The sexual distress scale is validated as a linear scale with higher scores indicating greater sexual distress. The value of analyzing separate questions is not validated [18, 21, 22]. The validated cut-off score of ≥ 15 indicates the presence of sexually related personal distress [22]. The SDS asks for topics such as regret, dissatisfaction and frustration with sex life. The BIS was developed to assess changes in body image in patients diagnosed with all types of cancer. The BIS is also validated in other diseases such as inflammatory bowel disease [23], making it a suitable PROM for comparison between disease groups The BIS contains ten questions which have to be answered on a 4-point Likert scale, with higher scores indicate higher body image concerns [17, 19, 24]. As five questions of the scale consider the effect of a disease or treatment on body image, these questions were not analyzed for the respondents, resulting in analyses of non-disease related questions 1, 3, 5, 7 and 9, resulting in a maximum score of 15. Both PROMs are proven to be valid and reliable with acceptable internal consistency and test–retest reliability [17–19, 21, 22, 24].

### Distribution and population

We distributed the validated Dutch translation of the SDS, BIS and additional demographic questions on gender (male, female and non-binary), age, education level, relationship status, (psychological) comorbidities and history of cancer via ‘’Limesurvey’’ via two ways. First, the members of the research team disseminated the hyperlink to friends and family via social media and asked them to distribute it to others, creating a snowballing effect. Second, the hyperlink was distributed via the official LinkedIn and Facebook accounts of the Erasmus University Medical Center. Participants could participate if they were ≥ 18.

Informed consent was retrieved after notification of study information. Participants were not compensated and could withdraw at any moment. Missing data was handled on a survey by survey basis, meaning that respondents who finished the SDS but not the BIS were included in the analyses of the SDS but not the BDS. Incomplete questionnaires were used for non-responders analyses.

Data was collected between 12-05-2022 and 08-08-2022. The Dutch Medical Research Act did not apply to this study, which was confirmed by the local Medical Ethics Review Committee (MEC-2022-0154).

### Statistical analyses

Response data was analyzed using post-stratification cell-based weighting. The sample was weighted by sex, age (18–24, 25–35, 35–45, 45–55, 55–65, > 65) and education level (low, middle, high educational level) based on the Dutch population distribution in August 2022 [25]. The Charlson Comorbidity Index (CCI) was calculated based on the comorbidities reported in the questionnaire by respondents.

Respondents who did not finish all questionnaires but submitted demographic data (i.e., age and gender) were included in the non-responder analyses. The independent T-test and the Fisher`s exact test were used for age and gender respectively.

For both scales descriptive statistics were calculated to present the normative data per age group per gender. For the Sexual Distress Scale we used the cut-off score of ≥ 15 to conduct a multivariable logistic regression to assess the effect of age, gender, education, relationship status, history of cancer, Charlson Comorbidity Index score [26] (CCI) and psychological comorbidities on the outcome of sexually related personal distress.

To assess the effect of demographic variables on the scores of the non-disease related questions of the BIS, we conducted a multiple linear regression to assess the effect of age, gender, education, relationship status, history of cancer and CCI and psychological comorbidities on the sum of the five non-disease related questions. The residuals were tested on normality and homoscedasticity with residual plots. P-values < 0.05 were considered statistically significant. Statistical analysis was performed using SPSS, Version 28.0.1.0 [27].

## Results

### Study participants

The survey was opened 956 times, of which 772 (80.8%) respondents completed the SDS and demographic variables. The BIS was completed by 698 (73.0)% respondents. As the numbers of non-binary responders were small (n = 3 for the SDS, n = 2 for the BIS), these responders were excluded from the analyses of both scales, resulting in 769 respondents for the SDS and 696 respondents for the BIS. The number of patients per gender per age group before weighting is presented in supplementary table 1.

For the non-responders analyses we compared the complete responses (n = 698) with the incomplete responses (n = 175). The non-responders were older than the responders (p = 0.008). There was no evidence for a difference in gender (Table 1).

**Table 1:** Difference in demographic variables between responders and non-responders

	Responders	Non-Responders	p-value
	*Mean (SD), N (%)*	*Mean (SD), N (%)*	
Age	39.33 (13.70)	42.39 (15.39)	0.0079*
Gender			
Male	113 (16.2)	28 (16.0)	0.319
Female	583 (83.5)	145 (82.9)	
Non-binary	2 (0.3)	2 (1.1)	

SD = standard deviation

* p-value ≤ 0.05

### Sexual distress scale

#### Demographic variables

**Fig. 1:**
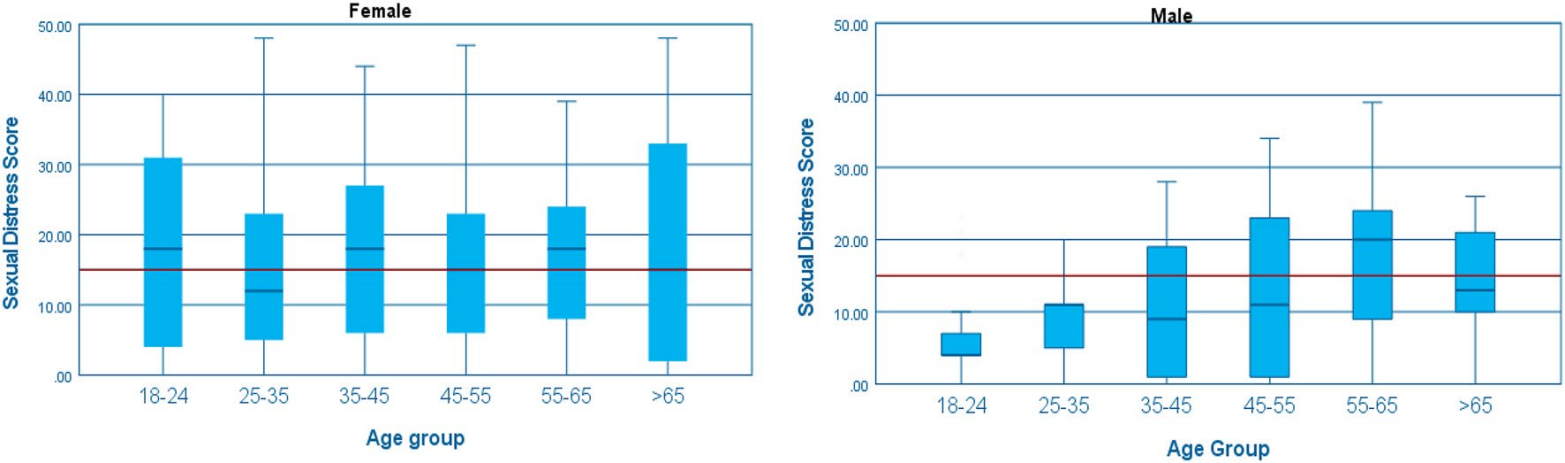
Weighted score of the Sexual Distress Scale per age group and gender. Presented in boxplot: Median, IQR and range and the reference line of personally sexual distress by a Sexual Distress Score ≥ 15

We included 769 respondents with a median age of 38.0 (IQR 27.0; 61.0) and mean SDS score of 14.41 (SD 10.98). The majority of respondents were female (84.1%), had completed a high educational level (n = 530, 68.9%) and 79.5% of the respondents had a CCI of 0 (Table 2). Compared to the Dutch population, male respondents and respondents with low educational level are underrepresented. Female respondents score higher on the SDS in every age group, except from age 55–65, as compared to male respondents (Fig. 1). For the exact weighted norm scores per age group and gender, see Supplementary Table 2. The Cronbach’s alpha of the SDS for the total population, male and female was respectively 0.95, 0.94 and 0.95.

**Table 2:** Demographic variables

	SDS (n = 769)	BIS (n = 696)
	Median (IQR), N (%)	Median (IQR), N (%)
Age	38.0 (27.0; 61.0)	37.0 (27.0; 50.0)
Gender		
Male	122 (15.9)	113 (16.2)
Female	647 (84.1)	583 (83.5)
Education		
Low educational level	94 (12.2)	81 (11.6)
Middle educational level	145 (18.9)	132 (19.0)
High educational level	530 (68.9)	483 (69.4)
Relationship status		
Single	160 (20.8)	148 (21.3)
In a relationship	109 (14.2)	97 (13.9)
Living together	161 (20.9)	153 (22.0)
Married/Registered partnership	339 (44.1)	298 (42.8)
History of cancer		
No history of cancer	706 (93.9)	679 (97.6)
Cancer in the past	47 (6.1)	17 (2.4)
Psychological comorbidities		
Yes	49 (6.4)	46 (6.6)
Charlson Comorbidity Index		
0	611 (79.5)	545 (78.3)
1	104 (13.5)	101 (14.5)
≥ 2	54 (7.0)	50 (7.2)

IQR = Interquartile range

#### Multiple logistic regression of the weighted SDS score

Women had on average higher probability of experiencing sexual distress compared to men (OR 1.77, 95% CI [1.32; 2.39]). The odds of having personal sexual distress in people with a low educational level was higher (OR 2.02, CI [1.37; 2.39]) as compared to people with a middle educational level. Compared to single respondents, people who live together or are married have on average higher probability of experiencing of sexual distress with respectively an odds ratio of 2.27 (95% [1.35; 3.81]) and 1.93 (95% CI [1.26; 2.96]). The same holds for people with psychological comorbidities (OR: 4.86, 95% CI [2.17; 10.88]) versus no psychological comorbidities and respondents with a CCI ≥ 2 as compared to respondents with a CCI of 0 (OR: 1.86, 95% CI [1.20; 2.90]) (Table 3).

**Table 3:** Multiple logistic regression sexual distress scale

*Nagelkerke R*^*2*^ = *0.168*	Odds Ratio	95% CI	P-value
Age	1.001	0.990; 1.012	0.853
Gender			
Male	RF	RF	
Female	1.777	1.321; 2.391	< 0.001*
Education			
Low educational level	2.017	1.371; 2.968	< 0.001*
Middle educational level	RF	RF	RF
High educational level	0.728	0.518; 1.023	0.067
Relationship status			
Single	RF	RF	RF
In a relationship	0.919	0.541; 1.563	0.756
Living together	2.268	1.349; 3.812	0.0020*
Married/Registered partnership	1.929	1.256; 2.962	0.0027*
History of cancer			
No history of cancer	RF	RF	RF
Cancer in the past	1.712	0.984; 2.977	0.057
Psychological comorbidities			
Yes	4.857	2.167; 10.882	< 0.001*
Charlson Comorbidity Index			
0	RF	RF	RF
1	1.202	0.757; 1.911	0.436
≥ 2	1.861	1.196; 2.897	0.0059*

95% CI = 95% Confidence interval

*p-value ≤ 0.05

### Body image scale

**Fig. 2:**
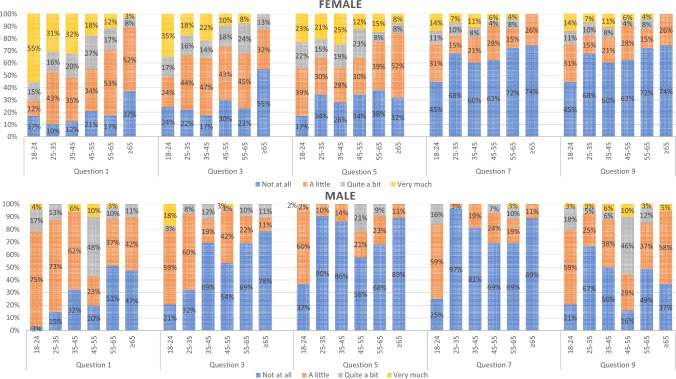
Weighted norm answers on non-disease related questions of the Body Image Scale per gender per age group. Percentages of the answers given by responders are presented per question. Q1 = Have you been feeling self-conscious about your appearance? Q3 = Have you been dissatisfied with your appearance when dressed? Q5 = Do you find it difficult to look at yourself naked? Q7 = Do you avoid people because of the way you feel about your appearance?

**Fig. 3:**
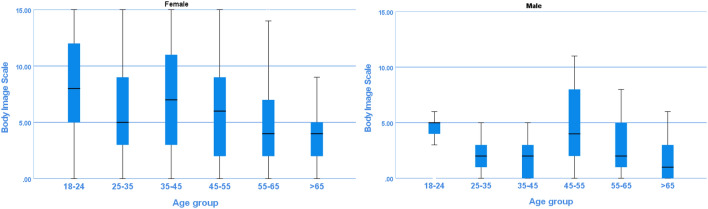
Weighted score of Body Image Scale per age group and gender. Presented in boxplot: Median, IQR and range

For the analysis of the BIS, 696 respondents were included, with a median age of 37.0 (IQR 27.0–50.0). The majority of respondents was female (83.5%) and finished higher education (69.4%) (Table 2). The weighted answers per question show that the percentage of female respondents who answer ‘’very much’’ are higher in question 1, 3, 5, 7 and 9 for every age group as compared to men/male respondents (Fig. 2). The weighted total score of the five questions is presented in Fig. 3. The exact weighted norm scores per age and gender are presented in supplementary table 3. The Cronbach’s alpha of the BIS for the total population, male and female was respectively 0.93, 0.93 and 0.94.

The multiple linear regression on the total score of non-disease related questions showed a positive association between psychological comorbidities (β: 2.45, 95% CI [1.43; 3.47]), female gender (β: 2.63, 95% CI [2.13; 3.13]) and the BIS score. Age (β: −0.07, 95% CI [−0.09; −0.05]), high educational level (β: −1.21, 95% CI [−1.79; −0.64]) and being in a relationship (β:−1.512, 95% CI [−2.39; −0.63]) were negatively associated with the BIS and thus with a better body image (Table 4).

**Table 4:** Multiple linear regression body image scale

*Adjusted R*^*2*^ = *0.243*	Beta (SE)	95% CI	P-value
Age	−0.073 (0.009)	−0.091; −0.054	< 0.001*
Gender			
Male	RF	RF	
Female	2.633 (0.254)	2.133; 3.132	< 0.001*
Education			
Low educational level	0.118 (0.328)	−0.525; 0.761	0.718
Middle educational level	RF	RF	RF
High educational level	−1.217 (0.294)	−1.794; −0.639	< 0.001*
Relationship status			
Single	RF	RF	RF
In a relationship	−1.512 (0.449)	−2.393; -0.631	< 0.001*
Living together	−0.598 (0.441)	−1.463; 0.267	0.175
Married/Registered partnership	−0.446 (0.359)	-1.150; 0.258	0.214
History of cancer			
No history of cancer	RF	RF	RF
Cancer in the past	0.505 (0.454)	−0.386; 1.395	0.266
Psychological comorbidities			
Yes	2.452 (0.522)	1.428; 3.476	< 0.001*
Charlson Comorbidity Index			
0	RF	RF	RF
1	0.481 (0.387)	-0.279; 1.242	0.214
≥ 2	0.733 (0.374)	0.000; 1.467	0.050

95% CI = 95% Confidence interval, SE = Standard Error

*p-value ≤ 0.05

## Discussion

This study describes normative values of the SDS, which are relatively high in all age groups, especially in women. Research has shown that women are more often insecure about their appearance how they look during sex and do things they do not always want to do [28]. Our results are also in line with normative scores for sexual distress in Australian women [15]. Moreover, it is known that body image, which is often lower in women, is associated with decreased sexual health [29]. On the other hand it can be, due to self-selection, that respondents with sexual problems are over represented in our study. As sexual function is associated with sexual distress it could explain the relative high SDS [13, 21]. Unfortunately this was not assessed in our study. In addition, the high normative scores of sexual distress in men in the age group of 55–65 could be caused by the increasing incidence of erectile dysfunction at this age [30].

This study demonstrates that gender, education level, relationship status and comorbidities are associated with personal sexual distress. This is in line with previous evidence that both relationship factors and psychosocial comorbidities such as depression are positively associated with sexual distress [11, 12, 15]. It is also known that less educated people in the Netherlands have less access to reliable information about sex and talk less about sex with friends, professionals or family. They also have more experience with sexual transgressive behavior and sexual violence, which can explain the association between low education level and more sexual distress [28, 30].

Body image is not only an essential factor for sexual health but also for HRQoL [4, 29]. In this study age and male gender were associated with a better body image. This is in line with earlier evidence that younger age and female gender are associated with body image disturbance [23, 31, 32]. Older people are inclined to accept their bodies more than teenagers do and are less concerned about weight and body shape [32, 33]. Female body image is extensively entwined with social ideals and norms, which may result in higher prevalence of body image disturbance [29]. Last, comorbidities, due to alternations in body compositions after for example surgery, are also known to impact body image [4, 6, 30, 34, 35].

A major strength of this study is the fact that it examines sexual distress rather than sexual function and dysfunction. There have been many studies that have focused on sexual function, even a study in the general Dutch population [5–8, 36–38]. None of them examine sexual distress, although it has been shown that sexual distress and sexual functioning are two different topics and are both important aspects of sexual health [3, 39]. Our study adds important data on normative scores for sexual distress in the general population. Other strengths are that this study has a large sample size, and reports both normative scores and evaluates demographic characteristics that may influence personal sexual distress and body image disturbance. Specialists can now compare the normative values of the Dutch population with the scores of their patients. This is of great value because it allows healthcare providers to provide patient-centered advice in practice.

A limitation of our study is the presence of both selection and responder bias. This is partly due to self-selection (as with most survey studies) and has resulted in the overrepresentation of young and highly educated women. It was not possible to complete a full non-responder analysis, because it is unknown how many people were reached with the survey link. Therefore, we conducted a non-responder analysis with incomplete responders, who answered at least the first questions on age and gender but did not complete the full survey. The results indicate that older people are more likely to stop halfway, possible because they have less interest in discussing this topic. The sample size in older age groups is also substantially lower than in younger groups probably because a web-based questionnaire was used. Older people may have less access to online platforms and social media. As a result, the original normative values of our data were initially not representative of the general Dutch population. To reduce responder and selection bias, post stratification cell-based weighting was applied. The data was converted based on the distribution of the general population in the Netherlands. It considers hard-to-reach demographic groups and therefore improves the representativeness of the sample. However, there may still be some bias because of the differences in sample size across the age groups. Smaller subgroups, mostly consisting of older people and men, may be generalized due to the survey weighing. The subgroups of men with a low level of education between the ages of 25 and 35 and men with a middle level of education between the ages of 65 and 75 are not present in our data. As such, it was not possible to include them in the analysis. More research is needed for the normative data of these two subgroups. Moreover, we asked for gender in our survey and stratified for sex as no data on gender was available for the Dutch population. However, we expect little difference between gender and age in our cohort, as the estimate of transgenders in the Dutch population is between 0.5 and 3% [40]. Moreover, as we did not assess the sexual dysfunction, we do not know whether there is an overrepresentation of people with sexual dysfunction in our sample. This could have affected our results, as sexual dysfunction is related with sexual distress ^(13,21)^.

Another challenge in this study were the disease-related questions of the BIS. As most responders reported no comorbidities (78%) the disease-related questions were not relevant. For this reason, only the non-disease related questions were used for analyses. As this is not the full body image score, this cannot be used for one-on-one comparison in the consultation room. However, it gives an indication which variables affect the outcome of the Body Image Scale regardless of the disease involved and should be taken into account when assessing the full Body Image Scale.

Last, we did not ask whether respondents had intercourse in the last four weeks, the recall period of the questionnaire. It would be interesting to see whether people with no intercourse could also experience sexual distress or whether this is only limited with people with (regular) intercourse.

### Further research

In the future more detailed insights on the effect of religion, sexual orientation, income and sexual attitudes should be examined by conducting in-depth interviews with in the general population. Moreover, the effect of sexual dysfunction on distress should be assessed. These will give more insights in how sexual health is effected and, in a later stage, how this can be improved. Finally, it is relevant to study the impact of chronic illness and cancer on sexual health to improve personalized medicine.

## Conclusion

This study provides age- and gender-dependent normative values for the Sexual Distress Scale and the non-disease related questions of the Body Image Scale to enable future comparisons in sexual health between Dutch patients and their age- and gender matched peers. Sexual distress and body image are influenced by sex, education level, relationship status and psychological comorbidities. Moreover, age is negatively associated with the Body Image Scale.

## Supplementary Information

Below is the link to the electronic supplementary material.

Supplementary file1 (PDF 449 KB)

## Data Availability

The data that support the findings of this study are available from the corresponding author upon reasonable request.
