# Supplementary material for: Dutch normative data of the sexual distress scale and the body image scale
Source: Qual Life Res. 2023 May 16;32(10):2829–37. doi: 10.1007/s11136-023-03434-w (PMC10473982; doi:10.1007/s11136-023-03434-w)
Supplement: Supplementary file 1 — Supplementary file1 (PDF 449 KB) [file 11136_2023_3434_MOESM1_ESM.pdf]

Journal: Quality of Life Research

Dutch normative data of the Sexual Distress Scale and the Body Image Scale

Anouk S. Huberts MD[1], Noëlle .J.M.C. Vrancken Peeters[2], Z.L. Rana Kaplan MD[3], Reinier C.A. van Linschoten MD[4,5], H. Pastoor[6], C. Janneke van der Woude MD PhD[5], Linetta B. Koppert MD PhD[1,2]

[1]Department of Quality and Patientcare, Erasmus University Medical Center, Rotterdam, The Netherlands.

[2]Academic Breast Cancer Center, Department of Surgical Oncology, Erasmus MC Cancer Institute, Erasmus University Medical Center, Rotterdam, The Netherlands

[3]Department of Public Health, Erasmus University Medical Center, Rotterdam, The Netherlands

[4]Department of Gastroenterology & Hepatology, Franciscus Gasthuis & Vlietland, Rotterdam, The Netherlands

[5]Department of Gastroenterology & Hepatology, Erasmus Medical Center, Rotterdam, The Netherlands,

[6] Division of Reproductive Medicine, Department of Obstetrics and Gynecology, Erasmus University Medical Center, Rotterdam, The Netherlands

Corresponding Author:

Anouk Huberts MD

E-mail: a.huberts@erasmusmc.nl

Supplementary table 1: Number of responders per age group per gender

	Sexual Distress Scale		Body Image scale	
	**Male** N(%)	**Female** N(%)	**Male** N(%)	**Female** N(%)
**Overall**	122 (15.9)	647 (84.1)	113 (16.2)	583 (83.5)
**18-25**	13 (1.7)	86 (11.1)	13 (1.9)	77 (11.1)
**25-35**	25 (3.3)	207 (26.9)	24 (3.4)	198 (28.4)
**35-45**	15 (2.0)	136 (17.7)	13 (1.9)	126 (18.1)
**45-55**	31 (4.0)	114 (14.8)	29 (4.2)	101 (14.5)
**55-65**	23 (3.0)	82 (10.7)	20 (2.9)	62 (8.9)
**>65**	15 (2.0)	22 (2.9)	14 (2.0)	19 (2.7)

Supplementary table 2: Sexual Distress Scale Score per age group and gender after weighting

	**Male**		**Female**	
	Median (IQR)	Mean (SD)	Median (IQR)	Mean (SD)
**Overall**	11.0 (4.0 ; 21.0)	12.4 (9.5)	16.0 (5.0 ; 26.8)	16.8 (12.4)
**18-25**	4.0 (4.0 ; 7.0)	7.9 (7.1)	18.5 (4.0 ; 31.0)	17.9 (13.0)
**25-35**	11.0 (5.0 ; 11.0)	10.2 (6.4)	12.4 (5.0 ; 23.0)	15.0 (11.5)
**35-45**	9.0 (1.0 ; 19.0)	11.1 (9.7)	18.0 (6.0 ; 27.2)	17.6 (11.9)
**45-55**	11.0 (0.7 ; 23.0)	12.0 (10.3)	15.0 (6.0 ; 24.3)	16.1 (12.3)
**55-65**	20.0 (9.0 ; 24.0)	18.5 (10.4)	18.0 (8.0 ; 24.2)	16.5 (10.4)
**>65**	13.0 (10.0 ; 21.0)	15.2 (6.9)	15.0 (2.0 ; 33.0)	17.7 (15.5)

Supplementary table 3: Body Image Scale Score of sum score of questions 1,3,5,7, and 9 per age group and gender after weighting

	**Male**		**Female**	
	Median (IQR)	Mean (SD)	Median (IQR)	Mean (SD)
**Overall**	4.0 (2.0 ; 7.0)	4.6 (3.9)	5.0 (2.9 ; 9.0)	6.0 (4.4)
**18-25**	5.0 (4.0 ; 9.0)	6.5 (4.1)	8.4 (4.4 ; 12.0)	8.0 (4.9)
**25-35**	3.0 (2.0 ; 7.0)	4.3 (4.0)	5.0 (2.9 ; 9.0)	6.2 (4.5)
**35-45**	3.0 (1.0 ; 8.0)	4.6 (4.3)	7.0 (3.0 ; 11.0)	7.0 (4.6)
**45-55**	4.8 (2.0 ; 8.0)	5.2 (3.7)	5.9 (2.0 ; 9.0)	5.7 (4.1)
**55-65**	3.0 (1.0 ; 5.0)	3.9 (3.5)	4.0 (2.0 ; 7.0)	5.1 (4.0)
**>65**	3.0 (1.0 ; 5.0)	3.1 (2.6)	4.0 (2.0 ; 5.0)	3.8 (2.7)
